# Navigating new norms: Addiction specialists' perspectives on opioid use disorder treatments and policy challenges in the fentanyl era

**DOI:** 10.1111/ajad.13653

**Published:** 2024-10-04

**Authors:** Jeremy Weleff, Nicholaus J. Christian, James X. Wang, Mohit Singh, Joao P. De Aquino, Andrew J. Saxon, Gabriela Garcia Vassallo

**Affiliations:** ^1^ Department of Psychiatry Faculty of Medicine & Dentistry, University of Alberta Edmonton Alberta Canada; ^2^ Department of Psychiatry Yale University School of Medicine New Haven Connecticut USA; ^3^ Neuroscience and Mental Health Institute University of Alberta Edmonton Alberta Canada; ^4^ Office of the Clinical Director National Institute on Drug Abuse Intramural Research Program, National Institutes of Health Baltimore Maryland USA; ^5^ Division of Hospital Medicine Johns Hopkins Bayview Medical Center, Johns Hopkins University School of Medicine Baltimore Maryland USA; ^6^ British Columbia Centre on Substance Use Vancouver Canada; ^7^ Division of Adolescent Health and Medicine University of British Columbia Vancouver Canada; ^8^ Department of Psychiatry University of Alberta Edmonton Alberta Canada; ^9^ VA Connecticut Healthcare System West Haven Connecticut USA; ^10^ Clinical Neuroscience Research Unit Connecticut Mental Health Center New Haven Connecticut USA; ^11^ Department of Psychiatry and Behavioral Sciences University of Washington School of Medicine Seattle Washington USA; ^12^ Center for Excellence in Substance Addiction Treatment and Education, VA Puget Sound Health Care System Seattle Washington USA

## Abstract

**Background and Objectives:**

Amidst increasing opioid‐related overdoses in the USA, opioid use disorder (OUD) treatment has seen few novel treatments emerge. High‐potency synthetic opioids (HPSOs) have altered clinical approaches, prompting evaluation of existing medications for opioid use disorder (MOUD) and interest in slow‐release oral morphine (SROM) as another therapeutic option. Here we survey addiction specialists on the influence of HPSOs on clinical practice, views on current MOUD regulations, and openness to novel therapies such as SROM.

**Methods:**

Anonymous, online survey conducted at a national conference of addiction specialists (*N* = 91). Pearson χ^2^ tests and Fisher's exact tests to compare respondent characteristics.

**Results:**

Approximately 89% of respondents (*N* = 91) acknowledge that HPSOs shifted addiction treatment in recent years, with 86% modifying their MOUD prescribing accordingly. Moreover, 84% report having patients who could benefit from other full opioid agonists beyond methadone for OUD management. Many report off‐label prescribing of full agonist opioids other than methadone for withdrawal symptoms or initiating MOUD. Eighty percent reported being in favor of incorporating SROM as a third‐line monotherapy for OUD.

**Discussion and Conclusion:**

This sample of addiction specialists supports innovative alternatives for MOUD in the USA to combat the challenges posed by fentanyl and related HPSOs. Future work should further addiction specialists' opinions on barriers to OUD treatment and exploration of these international strategies in the USA.

**Scientific Significance:**

This appears to be the first study exploring addiction specialists' perspectives on regulatory barriers to OUD treatment and their willingness to uptake internationally adopted strategies such as SROM.

## INTRODUCTION

The US drug overdose epidemic reached a grim milestone in 2021 with over 100,000 fatalities, highlighting the urgency to adapt clinical practices to the evolving threat posed by fentanyl and other high‐potency synthetic opioids (HPSOs).^1^, ^2^ In this context, there have been many calls to action to address the regulations surrounding the use of medications for opioid use disorder (MOUD) and improve access to these life‐saving medications.^3^, ^4^ During the COVID‐19 pandemic, the USA made incremental yet meaningful policy adjustments, such as easing methadone distribution through opioid treatment programs (OTPs) and abolishing the DATA waiver (X‐waiver), with the aim to expand access to MOUD. These measures represent strides towards aligning US policies with global standards for MOUD accessibility, yet the adoption of new treatment modalities has been slower than in some peer countries.

Research on addiction specialists' perspectives regarding fentanyl's influence on clinical practice, their assessment of current MOUD approaches, and their stance on the regulatory frameworks governing MOUD use is scant. In the USA, MOUD treatment predominantly relies on methadone, buprenorphine, and naltrexone. However, other treatment options are available internationally for those who do not benefit from these standard treatments. This discrepancy stems from the long‐standing effects of the Harrison Narcotics Act of 1914 and subsequent federal regulations, which constrict the range of opioid agonist medications permitted for OUD treatment.^5^ As an example, slow‐release oral morphine (SROM) is a 24‐h slow‐release formulation of morphine which, since at least the early 2000s, has seen a growing evidence base in its use as a therapeutic agent for OUD.^6^, ^7^, ^8^, ^9^, ^10^, ^11^, ^12^, ^13^, ^14^ SROM is consistently recognized or recommended in international treatment guidelines for OUD and in published strategies for MOUD.^15^, ^16^, ^17^, ^18^ Systematic reviews and meta‐analyses of randomized trials have substantiated SROM's efficacy, which is generally on par with methadone and buprenorphine in terms of treatment retention and reduction of opioid use, with a favorable safety profile.^6^, ^14^, ^19^ Additional data suggest that SROM may be particularly beneficial for individuals who have not responded to available first‐line treatments, experienced side effects (such as sedation from methadone), or have comorbid chronic pain.^7^, ^8^, ^13^ In addition, SROM can be used during transitions from methadone to buprenorphine or during methadone initiations,^15^, ^17^ and thus has potential as a useful tool in OUD care.

Investigating the potential integration of new treatment options for OUD is imperative for an effective response to the complexities of opioid use and OUD in our era. In this study, we explore the perspectives of addiction specialists in the USA on how HPSOs have impacted clinical practice, their views on current MOUD regulations, and their openness to adopting alternative MOUDs like SROM.

## METHODS

### Respondents and procedures

In April of 2023, we conducted an anonymous digital survey of addiction specialists in the USA who were attending the American Society of Addiction Medicine (ASAM) annual conference. Specifically, before the delivery of educational content, the survey was offered to attendees at the beginning of a workshop titled “Slow‐release oral morphine as MOUD: used internationally but why not the USA?”. All attendees of the workshop were considered eligible survey respondents. Respondents were provided with a QR code before the session and submitted answers electronically. Study data were collected and managed using Qualtrics and offered in English. The study was granted exempt approval by the Yale Institutional Review Board (IRB).

### Survey development

The survey, informed by a literature review of SROM and changing regulations regarding the provision of MOUD, intended to collect the following data: (1) practice characteristics of the responding clinician; (2) the clinician's experience and current MOUD practices; (3) their attitudes and beliefs regarding the impact of fentanyl on clinical practice; (4) and their willingness to prescribe full opioid agonists beyond methadone. G.G.V., J.W., and N.C. developed the survey instrument and the questions were iteratively refined by the workshop hosts.

### Measures

Demographics, including age, gender identity, race, ethnicity, profession, time in clinical practice, use of MOUD in clinical practice, and practice setting details, were collected. The full list of collected demographics is reported in Table 1. Responses to gather respondent opinions were measured using a five‐point Likert scale. Responding “somewhat agree” or “strongly agree” were considered favorable responses. Opinion statements were generally phrased such as: “*Fentanyl has greatly changed my addiction treatment practice in the last 5 years”* (Table 2). Two multiple‐choice follow‐up questions were asked if the respondent viewed the use of SROM favorably. Favorable responses were followed up with a question asking the specific applications in which clinicians would use SROM using branching logic.

**Table 1:** Participant characteristics.

		Currently prescribes full agonist opioids?		
	Total *n* = 82 (%)	No *n* = 57 (%)	Yes *n* = 25 (%)	*p*‐Value (α < .05)
Age				
25–34	19 (23)	12 (21)	7 (28)	.387
35–44	24 (29)	16 (28)	8 (32)	
45–54	18 (22)	11 (19)	7 (28)	
55–64	12 (15)	10 (18)	2 (8)	
65–74	9 (11)	8 (14)	1 (4)	
Gender identity				
Male/man	42 (51)	32 (56)	10 (40)	.288
Female/woman	38 (46)	23 (40)	15 (60)	
Transgender/nonbinary	2 (2)	2 (4)	0 (0)	
Race				
White	57 (70)	41 (72)	16 (64)	.702
Asian	17 (21)	10 (18)	7 (28)	
Black	3 (4)	2 (4)	1 (4)	
Other	3 (4)	2 (4)	1 (4)	
American Indian/Native American or Alaska Native	1 (1)	1 (2)	0 (0)	
Prefer not to say	1 (1)	1 (2)	0 (0)	
Hispanic or Latino(a)				
Yes	5 (6)	5 (9)	0 (0)	.052
No	77 (94)	52 (91)	25 (100)	
Profession				
MD/DO or equivalent	69 (84)	46 (81)	23 (92)	.436
Nurse Practitioner/Physician Assistant or equivalent	6 (7)	5 (9)	1 (4)	
Therapist	3 (4)	3 (5)	0 (0)	
Other	3 (4)	2 (4)	1 (4)	
Nurse	1 (1)	1 (2)	0 (0)	
Do you prescribe medications for opioid use disorder?				
Yes	75 (91)	51 (89)	24 (96)	.299
No	7 (9)	6 (11)	1 (4)	
Practice setting				
Outpatient only^a^	*n* = 38 (46)	41 (72)	5 (20)	**<.001**
Some inpatient^b^	*n* = 44 (54)	16 (28)	20 (80)	
Patient population				
Mostly urban	44 (54)	29 (51)	15 (60)	.275
Mostly rural	11 (13)	8 (14)	3 (12)	
Mix	17 (21)	12 (21)	5 (20)	
Veteran only	2 (2)	2 (4)	0 (0)	
Other unspecified	8 (10)	6 (11)	2 (8)	
Years in practice				
Less than 1 year	10 (12%)	5 (9)	5 (20)	.523
1–4 years	18 (22%)	12 (21)	6 (24)	
5–9 years	17 (21%)	14 (25)	3 (12)	
10–14 years	14 (17%)	10 (18)	4 (16)	
15+ years	23 (28%)	16 (28)	7 (28)	

*Note*: Bold value indicates statistically significant at *p* < .05.

^a^ *Outpatient only practice setting responses included*: Outpatient Addiction Specialty Clinic (*n* = 20), Outpatient Addiction Specialty Clinic and Primary Care (*n* = 7), Primary Care Only (*n* = 7), Outpatient Addiction Specialty Clinic and Psychiatry (*n* = 2), Outpatient General Psychiatry Only (*n* = 1), and Residential Treatment Program Only (*n* = 1).

^b^ *Some inpatient practice setting responses included:* Combination of Inpatient and Outpatient Addiction Settings (*n* = 20), Inpatient and Outpatient Addiction Medicine (*n* = 13), Addiction Consult Service and Primary Care (*n* = 4), Addiction Consult Service Only (*n* = 3), Inpatient Medicine and Primary Care (*n* = 2), and Inpatient Medicine Only (*n* = 2).

**Table 2:** Attitudes and beliefs of addiction providers regarding fentanyl and slow‐release oral morphine.

	*n* = 82 (%)
Fentanyl has greatly changed my addiction treatment practice in the last 5 years.	
Strongly agree	49 (60)
Somewhat agree	24 (29)
Neither agree nor disagree	8 (10)
Somewhat disagree	0 (0)
Strongly disagree	1 (1)
I prescribe MOUD differently due to the increase of fentanyl in the drug supply.	
Strongly agree	46 (56)
Somewhat agree	25 (30)
Neither agree nor disagree	7 (9)
Somewhat disagree	2 (2)
Strongly disagree	2 (2)
Current regulations interfere with my ability to provide the best evidence‐based treatments for my patients.	
Strongly agree	51 (62)
Somewhat agree	20 (24)
Neither agree nor disagree	6 (7)
Somewhat disagree	4 (5)
Strongly disagree	1 (1)
If practice standards included the use of full opioid agonists (outside of methadone) to treat opioid use disorder, I have patients that would benefit.	
Strongly agree	49 (60)
Somewhat agree	20 (24)
Neither agree nor disagree	12 (15)
Somewhat disagree	1 (1)
Strongly disagree	0 (0)
If practice standards included the use of SROM for the treatment of OUD, I would integrate it into my practice.	
Strongly agree	41 (50)
Somewhat agree	25 (30)
Neither agree nor disagree	16 (20)
Somewhat disagree	0 (0)
Strongly disagree	0 (0)
In my practice setting, I currently use off‐label full opioid agonists (other than methadone) to treat opioid withdrawal and/or to assist in starting medications for opioid use disorder?	
Yes	25 (30)
No	57 (71)
In what applications would you use slow‐release oral morphine for patients with opioid use disorder? (select all that apply)	*n* = 186 total responses (% reflects *n* = 82 respondents^a^)
Medically managed withdrawal	49 (60)
Third‐line mono‐treatment for OUD	66 (80)
To assist in methadone initiation	32 (39)
To treat pain	32 (39)
Other	7 (9)

Abbreviations: MOUD, medications for opioid use disorder; OUD, opioid use disorder.

^a^ Responses not mutually exclusive.

### Statistical analysis

We characterized the sample using descriptive statistics. We used Pearson χ^2^ tests and Fisher's exact tests to compare respondent characteristics who currently prescribe full agonist opioids to respondents who do not currently prescribe full agonist opioids. Practice setting was divided into “outpatient only” and “some inpatient” for the purposes of analysis given the sample size and heterogeneity of practice settings reported. *p* Values from these analyses are reported in Table 1. All analyses were conducted using IBM SPSS Statistics.

## RESULTS

### Respondent characteristics

Of the estimated 150 workshop attendees, as determined by attendance head count, 91 attendees completed the survey yielding an approximate 60.6% response rate. Nine respondents indicated that they practiced in Canada; thus, these responses were excluded from the analysis, which focused on this group of US addiction specialists' perspectives.

Ages were well distributed across respondents and there were almost equal numbers of those identifying as male/man as female/woman (51%–46%). Most respondents were White (70%), and only 6% identified as Hispanic or Latino(a). Of those who completed the survey, the majority were healthcare professionals with prescribing capacity, 84% were physicians (MD or DO) and 7% were either nurse practitioners or physician assistants (NP or PA). Ninety one percent of all respondents reported prescribing MOUD in their clinical practice. Most (54%) practice settings included some inpatient work, with 46% reporting exclusively outpatient work. Over half of respondents worked in mostly urban settings (54%), while 34% served patients from rural settings to some degree (13% mostly rural and 31% mixed rural and urban). Years in clinical practice were evenly distributed from less than 1 year to greater than 15 years. Table 1 details respondent characteristics.

### Perspectives on the impact of fentanyl on practices and willingness to adopt new treatments

Most respondents report that fentanyl/HPSOs greatly changed their practice of addiction treatment in the last 5 years (89%) or that they prescribe MOUD differently due to the presence of HPSOs in the drug supply (86%). A strong majority (86%) state the current regulations interfere with their ability to provide the best evidence‐based treatments for their patients. Additionally, 84% stated that if other full‐opioid agonists (other than methadone) were available to treat OUD, they have patients who would benefit. Eighty percent stated that if practice standards included the use of SROM for OUD, they would integrate it into their practice, with 60% stating they would prescribe it to mitigate opioid withdrawal, 80% stating they would use it as a third‐line monotherapy for OUD, and 39% stating they would prescribe SROM to assist with methadone initiations and/or treat pain.

Nearly one‐third (30%) of respondents reported using off‐label full opioid agonists to treat opioid withdrawal and/or assist with starting MOUD. Respondents who reported using off‐label full opioid agonists were more likely to practice in the inpatient setting compared to outpatient only practice (Pearson χ^2^ = 19.028, *p* < .001). There were no statistically significant differences in age, gender identity, race, profession, urban/rural patient population or years in practice between respondents who use off‐label full opioid agonists for OUD compared to providers who do not. Table 2 details participants' responses to questions about OUD regulations and integration of opioid agonists like SROM into OUD treatment.

## DISCUSSION

This survey provides insight into some US addiction specialists' views on MOUD regulations and their readiness to incorporate new treatments in response to widespread availability of HPSOs, which has escalated the opioid crisis. Results from our survey of MOUD prescribers, many of whom had over 10 years of clinical experience, suggest that this group of addiction specialists in the USA have changed their practices in response to shifts in the drug supply. The results here highlight this shift in clinical practice, an acknowledgment of the limitations imposed by current regulations, and a significant interest in additional treatments, particularly SROM. It is also in line with research on patient perspectives with the challenges of initiating treatment with fentanyl in the drug supply.^3^, ^20^ It is these challenges that have led to the recent updates of clinical guidelines to aid in the initiation of opioid agonist medications in the fentanyl era.^16^, ^21^

It is interesting to note that 30% of addiction specialists surveyed already use off‐label full opioid agonists to assist with starting MOUD and/or manage opioid withdrawal. It is not surprising that these healthcare professionals are more likely to practice in the inpatient setting, which is a practice setting explicitly mentioned in Title 21 CFR §1306.07 (the “3 Day Rule”), which allows for time‐limited dispensing of “narcotic drugs” for maintenance or detoxification while other treatment arrangements are being made.^22^, ^23^ Prior US studies and reports have described utilizing short‐acting full opioid agonists, such as oral oxycodone or hydromorphone and intravenous hydromorphone to assist in withdrawal management in the inpatient setting.^24^, ^25^ These medications offer more predictable effects in both pharmacokinetics and pharmacodynamics compared to illicit fentanyl and HSPOs.^26^ It is, therefore, pharmacologically plausible that these full opioid agonists could enable a more seamless transition from illicit HPOs to buprenorphine therapy.^20^ It is also possible that addiction specialists are managing pain in patients undergoing opioid withdrawal, a common and challenging aspect of the condition, which may be exacerbated by illicit fentanyl exposure.^27^, ^28^ The controlled hospital environment may also alleviate safety concerns associated with the use of full opioid agonists (whether those concerns be oversedation, falls, diversion, etc). It is essential to report and monitor off‐label prescribing and patient safety needs. Our findings here imply that with any changes in federal regulations, inpatient providers may more rapidly integrate these adjustments into their clinical practice, necessitating a parallel increase in educational efforts for outpatient settings to keep pace with such developments.

The balancing of possible safety risks, cost‐effectiveness, and other unforeseen aspects of SROM implementation in the USA would require continued attention. SROM's utility would likely mostly be seen within the OTP setting in the USA, which allows for a controlled setting of monitored dosing, which should hopefully minimize the safety risks and other possible risk of medical profiteering. However, to date, the cost‐effectiveness of SROM in OUD care has not been determined due to a lack of studies addressing this question, particularly in the USA.^29^

The overwhelming majority of respondents reported an interest in utilizing SROM for the treatment of OUD, which is currently utilized as second or third‐line treatment in some European countries and Canada. This indicates that current regulations and MOUD alternatives in the USA may be impacting healthcare professionals' capacity to provide comprehensive care, as evidenced by more than 80% of survey respondents who believe their patients could benefit from a broader range of treatment strategies. Moreover, these findings highlight the need to evaluate the use of effective alternative MOUD, such as SROM, in the USA, as well as the current policies that impact addiction treatment. Additionally, the FDA has set out standards for real‐world data and real‐world evidence to support FDA regulatory decisions,^30^ for which the international experience of SROM may be well suited. Identifying strategies such as those utilized with methadone, which operated under Investigational New Drug before its eventual full regulation, is critical to the implementation of new treatments and the study of their efficacy. Embracing OUD prescribing practices aligned with growing international consensus may constitute an integral part of a larger, multi‐pronged strategy to combat the opioid crisis in the USA more effectively.

### Limitations

The results presented should be interpreted in the context of a nonrandom, self‐selected sample of addiction specialists who attended a major national academic conference. Notwithstanding their expertize, these specialists' responses might carry inherent biases due to their specific interest in SROM, as evidenced by their attendance at a related workshop, and their decision to participate in the survey. This may greatly limit generalizability to all who provide OUD care in the USA, but presents a starting point for further investigations. Additionally, the absence of qualitative data in our study limited our ability to capture nuanced insights into the complex attitudes and decision‐making processes of practitioners. In addition, the risk of non‐anonymity within a conference setting amongst a relatively small community of practice may limit fully sincere responses to a survey of this nature. Of course, this is only one study, and to obtain a comprehensive representation of opinions across the spectrum of US healthcare professionals who interact with those with OUD, such as pharmacists, as well as those with lived/living experience, future research should endeavor to include a more varied participant pool and incorporate qualitative methodologies for a more balanced analysis.

## CONCLUSION

US addiction specialists are adapting their practices in response to the opioid crisis, particularly the prevalence of HPSOs. The results here suggests that current MOUD regulations may restrict what is perceived to be optimal care for those with OUD in the USA, with a notable openness among professionals to integrate additional full‐opioid agonists into treatment regimens. Further exploration in larger, more diverse samples is warranted to assess the potential for regulatory reform and the adoption of new treatment modalities, such as SROM, in the USA. These efforts are essential to effectively address the complexities of OUD treatment in the modern US landscape.

## CONFLICT OF INTEREST STATEMENT

The authors declare no conflicts of interest.
